# TDP-43 and Phosphorylated TDP-43 Levels in Paired Plasma and CSF Samples in Amyotrophic Lateral Sclerosis

**DOI:** 10.3389/fneur.2021.663637

**Published:** 2021-06-14

**Authors:** Yuting Ren, Siyuan Li, Siyu Chen, Xiaosun Sun, Fei Yang, Hongfen Wang, Mao Li, Fang Cui, Xusheng Huang

**Affiliations:** ^1^Department of Neurology, First Medical Center, Chinese PLA General Hospital, Beijing, China; ^2^Department of Neurology, Beijing Tiantan Hospital, Capital Medical University, Beijing, China; ^3^Department of Geriatric Neurology, Second Medical Center and National Clinical Research Center for Geriatric Diseases, Chinese PLA General Hospital, Beijing, China; ^4^Department of Neurology, Tianjin Third Central Hospital, Tianjin, China

**Keywords:** amyotrophic lateral sclerosis, biomarker, TDP-43, pTDP-43, CSF, plasma

## Abstract

**Objective:** The aim of this study was to measure both plasma and cerebrospinal fluid (CSF) TAR DNA-binding protein 43 (TDP-43) and phosphorylated TDP-43 (pTDP-43) levels in sporadic amyotrophic lateral sclerosis (sALS) patients, and to compare them with that of healthy controls. The correlation between plasma or CSF TDP-43/pTDP-43 and clinical indicators of ALS patients was assessed.

**Methods:** Paired plasma and CSF TDP-43/pTDP-43 levels in 69 ALS patients and 59 healthy controls were measured by sandwich ELISA. Time to generalization (TTG), an indicator suggested that the time of symptoms spreading from spinal or bulbar localization to both, was evaluated in all patients screened for mutations in genes associated with ALS.

**Results:** Both of the plasma TDP-43 and pTDP-43 levels were significantly higher in ALS patients than HCs (*P* < 0.001). The pTDP-43/TDP-43 ratios in plasma were significantly higher in HCs than ALS patients (*P* < 0.001). The area under the curve (AUC) value was 0.924 for plasma TDP-43 level, with a 91.3% sensitivity and 91.5% specificity. Moreover, the correlation between plasma and CSF TDP-43 was observed in each ALS patient (*r* = 0.195, *P* = 0.027). A correlation between CSF pTDP-43 levels and the ALSFRS-R (*r* = −0.245; *P* = 0.042) was established. A correlation was observed between plasma TDP-43 levels and TTG in ALS patients, which indicated that high levels of plasma TDP-43 correlated with prolonged TTG (*r* = 0.415; *P* = 0.004).

**Conclusion:** The plasma TDP-43 and pTDP-43 levels might play an important role in diagnosis in the future study of ALS. The plasma TDP-43 might differentiate ALS and HC groups based on high sensitivity and specificity, and as an indicator of progression of disease.

## Introduction

Amyotrophic lateral sclerosis (ALS) is a progressive neurodegenerative disease characterized by the degeneration of both upper and lower motor neurons, with a median survival of 3–5 years (1). The pathogenesis of the disease is not fully elucidated and there is still lack of effective treatments. Thus, a specific and reliable biomarker is essential for diagnosis, assessment of disease progression and prognosis in ALS patients.

Previous reports indicated that plasma TDP-43 protein levels was elevated in 46% patients with frontotemporal dementia (FTD) as compared to the control subjects as assessed by ELISA (2). Interestingly, based on the autopsy studies, the proportion of FTD patients showing increased plasma TDP-43 levels was similar to that of patients known to harbor TDP-43 pathological changes in brains. These results concluded that increased biofluids such as plasma or cerebrospinal fluid (CSF) levels of TDP-43 may indicate the presence and degree of TDP-43 pathology within the brain (2). Although a range of antibodies can be used to detect TDP-43 in biofluids, an incredibly highly variability of detection in samples from ALS and FTD patients has been reported (3). Several studies explored the role of plasma and CSF TDP-43 levels as a biomarker in patients with FTD or ALS and demonstrated variable results (2, 4–8). Foulds et al. developed an ELISA-based method to assess the levels of phosphorylated TDP-43 (pTDP-43) within plasma samples, which seemed to be more reliable than total TDP-43 in distinguishing FTD-TDP from other forms of FTD or Alzheimer's disease (5). Noto et al. showed that the CSF TDP-43 levels were elevated only in ALS patients and lower CSF TDP-43 levels may be associated with worse survival (8). Steinacker et al. reported that patients with ALS and FTD had higher TDP-43 levels than controls in CSF, which indicated that CSF TDP-43 might aid in characterizing the subgroups of patients across the ALS and FTD disease spectrum (6). Suarez-Calvet et al. used ELISA to estimate the pTDP-43 levels in plasma and CSF in FTD patients and healthy controls, thereby suggesting that plasma pTDP-43 levels may be increased in subjects with mutations in *GRN* and *C9orf72* genes (9). Majumder et al. conducted a meta-analysis suggesting that CSF TDP-43 levels could be a promising biomarker in FTD-ALS spectrum disorders in particular ALS patients (10). Junttila et al. showed that higher CSF TDP-43 levels in ALS patients and this finding was independent of the *C9orf72* expansion carrier status, and suggested that CSF TDP-43 levels might be an indicator of a more rapid progression in ALS (11). Lower TDP-43 levels in CSF have also been observed in *C9orf72* repeat expansion carriers with underlying TDP-43 pathology and a shorter survival in ALS patients. Kasai et al. showed that increased levels of CSF TDP-43 in sALS compared with control groups. The elevated CSF TDP-43 levels may preempt the accumulation of TDP-43 pathology in the central nervous system, or parallel with TDP-43 pathology in the early stage thereby serving as a biomarker for ALS (4). The combined use of CSF neurofilament light chain (NfL) and CSF TDP-43 may be a useful biomarker for the diagnosis of ALS (12). Bourbouli et al. found that combined analysis of CSF TDP-43, total tau protein (τT), and tau protein phosphorylated at threonine 181 would be of help in the antemortem diagnosis of ALS (13). Hosokawa et al. reported that TDP-43 concentrations in CSF were significantly higher in ALS than in Guillain-Barré syndrome (GBS), which suggested that quantitative measurement of CSF TDP-43 levels is a potential laboratory testing for differentiating ALS from peripheral neuropathies (14). In a relatively large cohort of 219 sALS patients and 100 healthy controls, Verstraete et al. confirmed that TDP-43 plasma levels were significantly increased in ALS patients and found a positive correlation with age in all subjects (7). However, whether phosphorylation of TDP-43 is a useful biomarker or an early pathological event in disease, perhaps even promoting mislocalization and aggregation, or is secondary to degradation processes or aggregate formation stays to be elucidated (3). Therefore, the present study aimed to measure both plasma and CSF TDP-43 and pTDP-43 levels in ALS patients. Furthermore, the correlations between TDP-43/pTDP-43 levels and clinical indicators such as age, disease duration, amyotrophic lateral sclerosis functional rating scale revised (ALSFRS-R) score and disease progression rate were also examined. In addition, we applied an early clinical parameter of survival that previously reported, the time of symptoms spreading from spinal or bulbar localization to generalization, known simply as time to generalization (TTG) (15, 16).

## Methods

### Subjects

The study enrolled 78 patients admitted to the Department of Neurology, Chinese PLA General Hospital, from November 2013–May 2018, from ALS patients with definite or probable ALS according to the revised El Escorial criteria (17) diagnosed by a neurologist specialized in motor neuron diseases. All the ALS patients who participated in our study were apparently sporadic cases. A family history of dementia was excluded and in the main routine a comprehensive neuropsychological evaluation including Mini-Mental State Examination (MMSE), Montreal Cognitive Assessment (MOCA) and Frontal Assessment Battery (FAB) were conducted on all ALS patients, where no patient was found to have cognitive impairment. We also screened the ALS patients for mutations in *TARDBP, SOD1, FIG4, FUS, CHMP2B, VCP, ELP3, SETX, HNRNPA1, SPG11, NEFH, VAPB, ANG, OPTN, UBQLN2, SQSTM1, MATR3, ATXN2, PFN1* and *DCTN1* genes using multiplex PCR, variants identified in our study had been classified as pathogenic, likely pathogenic, uncertain significance, likely benign, or benign in accordance with *Standards and Guidelines for the Interpretation of Sequence Variants: A Joint Consensus Recommendation of the American College of Medical Genetics and Genomics and the Association for Molecular Pathology* and ClinVar database (http://www.ncbi.nlm.nih.gov/clinvar). Given the potential consequences of gene heterogeneity, nine patients with ALS carrying pathogenic variants were excluded (Supplementary Material 1). Lastly 69 ALS patients were recruited for this study. The present study was approved by the Ethics Committee of the Chinese PLA General Hospital. Informed consent was obtained from all patients and controls. The clinical indicators of the ALS patients were recorded, including age, age of onset, site of symptom onset, diagnostic category (clinically definite, clinically probable, clinically probable-laboratory-supported, or clinically possible ALS). The functional status of the ALS patients was rated using the ALSFRS-R (18), and the disease progression rate (DPR) was calculated based on the established formula: ΔFS = (48–ALSFRS-R score)/(disease duration from initial symptoms onset to evaluation in months) (19). TTG, an early clinical parameter of disease progression, was also assessed in all ALS patients (20).

The healthy control (HC) group comprised of 59 age-matched subjects who initially visited Neurology Clinic and underwent lumbar puncture for the purpose of making the exact diagnosis; however, the final diagnosis were free from neurological diseases.

### Plasma and CSF Collection and Processing

CSF and blood samples were collected at the time of diagnosis in our department for analysis as described previously (21). All samples were processed within 5h following collection and stored at −80°C until further use. CSF was collected into polypropylene tubes by lumbar puncture and blood was collected into ethylenediaminetetracetic acid (EDTA) tubes, followed by centrifuged at 3,000 rpm for 15 min (4°C) to remove the cells and debris.

### Total TDP-43 ELISA Assay

Total TDP-43 was determined in plasma and CSF using a commercially available ELISA kit (Human TDP-43, KE00005, Proteintech, Chicago, USA) according to the manufacturer's instructions. The standard provided by the manufacturer consisted of a His-tag recombinant human full-length TDP-43 protein (Cat. # ag13119, Proteintech). All the analysis were performed on coded samples by the analyst blinded to the patient data. Results based on duplicates with CV>10% were excluded. Each experiment was repeated at least twice. Plasma and CSF samples were tested undiluted with a single batch of reagents, respectively.

### Phosphorylated TDP-43 ELISA Assay

pTDP-43 levels in plasma and CSF were measured in duplicate by a commercially available ELISA kit (pTDP-43 ELISA Kit, E9442h EIAab, Wuhan, China) according to the manufacturer's instructions. In this assay, a biotinylated rabbit polyclonal antibody against TDP-43 phosphorylated at Ser409 [antiphospho-TDP-43 (pSer409), Cat. # SAB4200223 Sigma-Aldrich, Saint Louis, MI, USA] was used as a detection antibody. The standard provided by the manufacturer consisted of recombinant human TDP-43 phosphorylated at Ser409. The results were expressed as relative units (plasma or CSF), generated from concentration values normalized to a standard sample loaded on all plates as previously described (9). Results based on duplicates with CV>15% were excluded. The plasma and CSF samples were tested undiluted.

### Statistical Analyses

Median levels and interquartile range (IQR) were used to indicate the central value and range, respectively. The χ^2^ test was used to compare the differences in categorical variables, and Fisher's exact test was used to analyze the contingency tables, wherein the sample sizes were <5. Non-parametric statistics (Kruskal–Wallis and Mann–Whitney *post-hoc* test) were used because of the non-normal distribution and heterogeneity of variance of a majority of the variables and parametric statistics were determined by Spearman's rank correlation coefficient (*r*). Receiver operating characteristic (ROC) curve analysis was performed for evaluation in the ALS patients; the area under the curve (AUC) was calculated and optimal cut-off values were derived by Youden index. *P*-values were two-sided, and ≤ 0.05 were considered statistically significant. All analyses were performed using the SPSS for Windows (Version 21.0, IBM Corp., Armonk, NY, USA).

## Results

**Figure 1:**
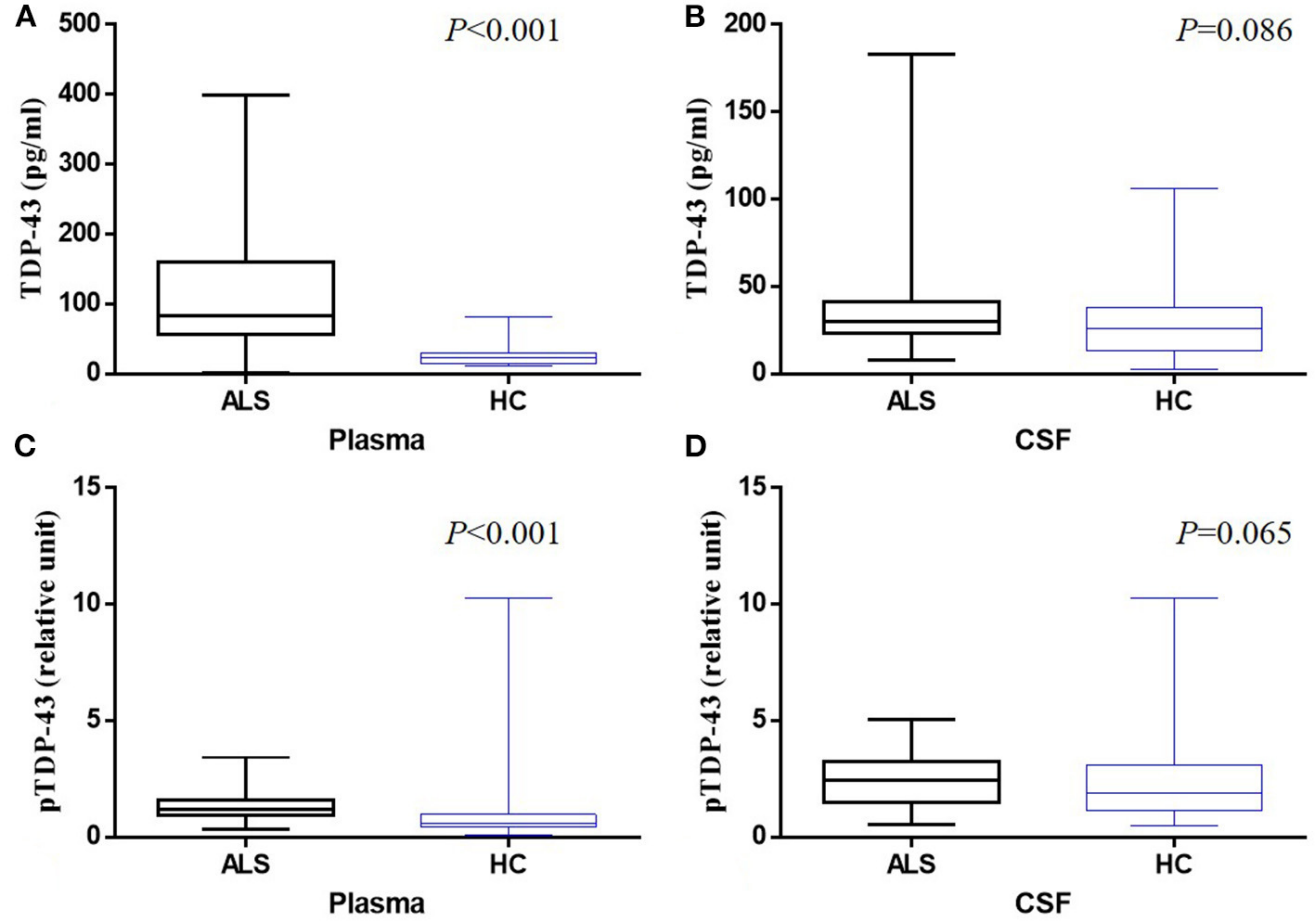
Box-whisker plots illustrate concentrations of TDP-43 in plasma **(A)** and CSF **(B)** and pTDP-43 in plasma **(C)** and CSF **(D)** from ALS patients and healthy controls (HC). The horizontal line in the box represents the median. Bottom and top of the box represent the 25^th^ and 75^th^ percentile, respectively. The whiskers (error bars) represent the range.

Sixty nine ALS patients were recruited for this study which comprised 46 males and 23 females with a mean (SD) age of 51.46 (9.42) years. The onset site was classified as spinal onset in 59 patients and as bulbar onset in 10 patients. The median average time from clinical onset to diagnosis was 19.59 months in ALS. The clinical characteristics of all participants are summarized in Table 1. The mean plasma level of TDP-43 was 113.10 and 25.19 pg/ml in ALS and HC groups, respectively; whereas, that in the CSF was 35.90 and 30.81 pg/ml, respectively. The mean pTDP-43 level in the plasma was 1.34 and 1.11% for ALS and HC groups, respectively, while the mean levels in CSF were 2.44 and 2.16%, respectively. The plasma TDP-43 level was significantly higher in ALS than HC (*P* < 0.001) (Figure 1A). There was no significant difference in CSF TDP-43 and pTDP-43 levels between ALS and HCs (*P* > 0.05) (Figures 1B,D). In addition, the plasma pTDP-43 level was also significantly higher in ALS than HC (*P* < 0.001) (Figure 1C). Apart from that, the pTDP-43/TDP-43 ratios in plasma were significantly higher in HCs than ALS patients (*P* < 0.001), but no significant difference was found in CSF (*P* = 0.409).

**Table 1:** Clinical characteristics of the participants.

	**ALS**	**HC**	***P*-value**
N (M/F)	69 (46/23)	59 (37/22)	0.640
Age (years)	51.46 (34–69)	51.76 (23–76)	0.353
Age of onset of the disease (years)	49.84 (30.50–68.25)	–	–
Site of onset (bulbar/spinal)	10/59	–	–
Categories at presentation of ALS	51/18	–	–
(definite/probable)			
Disease duration (months)	19.59 (3–60)	–	–
TTG (months)	12.03 (1–33)	–	–
ALSFRS-R	37.91 (18–47)	–	–
Progression rate	0.71 (0.04–2.67)	–	–

*Data are expressed as mean (range). ALS, amyotrophic lateral sclerosis; HC, healthy control; TTG, the time to generalization; ALSFRS-R, amyotrophic lateral sclerosis functional rating scale revised*.

**Figure 2:**
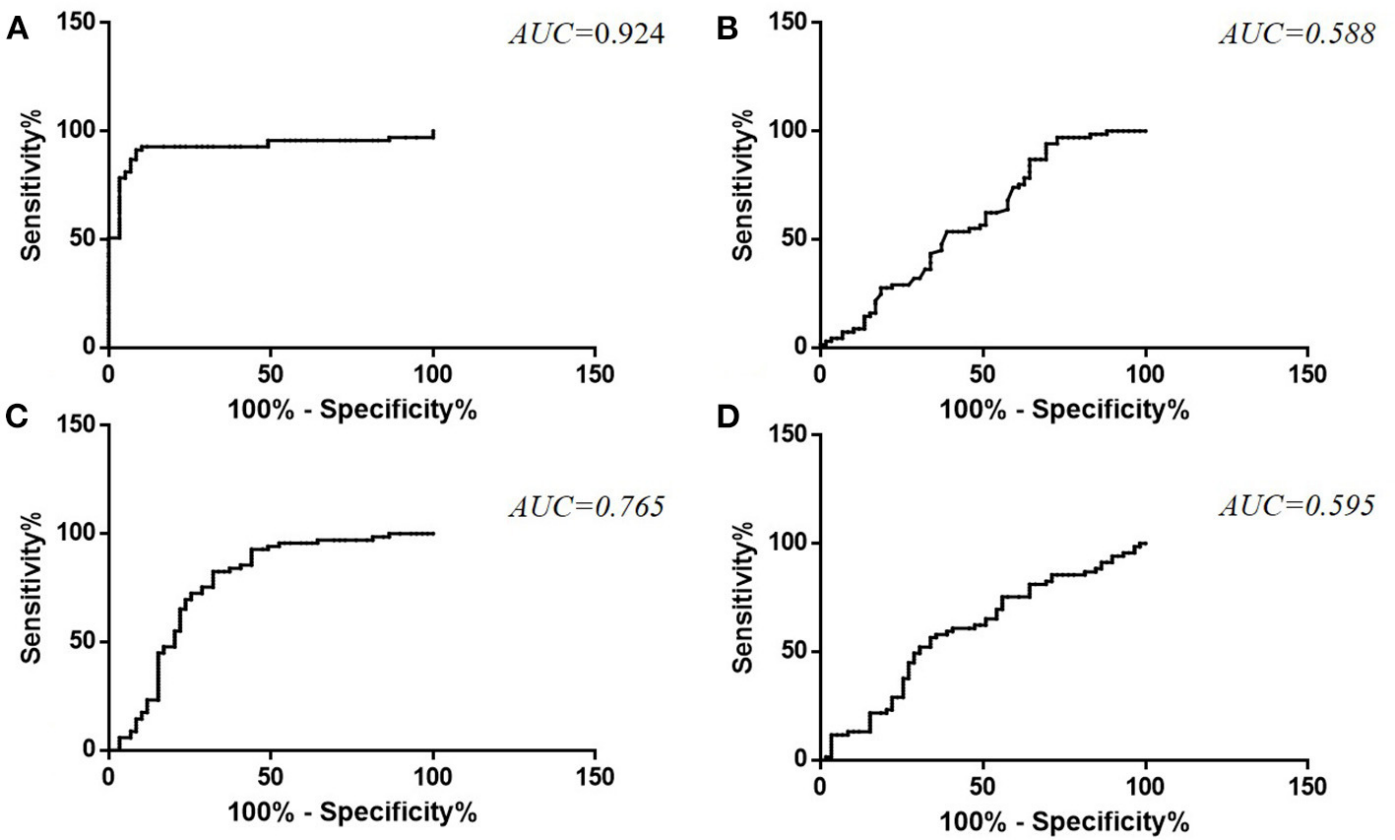
ROC curve for TDP-43 levels in plasma **(A)** and CSF **(B)** and pTDP-43 levels in plasma **(C)** and CSF **(D)** in ALS patients as compared to healthy controls.

ROC curve analysis was used to differentiate ALS from HC, and the AUC values were 0.924 and 0.588 for plasma and CSF TDP-43 levels, respectively. A cut-off level of 37.58 pg/ml for TDP-43 in plasma generated a sensitivity of 91.30% and specificity of 91.50% (Figure 2A). The optimal cut-off value for TDP-43 levels in CSF (15.08 pg/ml) generated a sensitivity of 94.20% and specificity of 30.50% (Figure 2B). The AUC values were 0.765 and 0.595 for plasma pTDP-43 levels and CSF pTDP-43 levels, respectively. A cut-off level of 0.86% for pTDP-43 in plasma provided a 82.60% sensitivity and 67.80% specificity (Figure 2C). The optimal cut-off value for pTDP-43 levels in CSF (2.31%) provided a 56.50% sensitivity and 66.10% specificity (Figure 2D).

**Figure 3:**
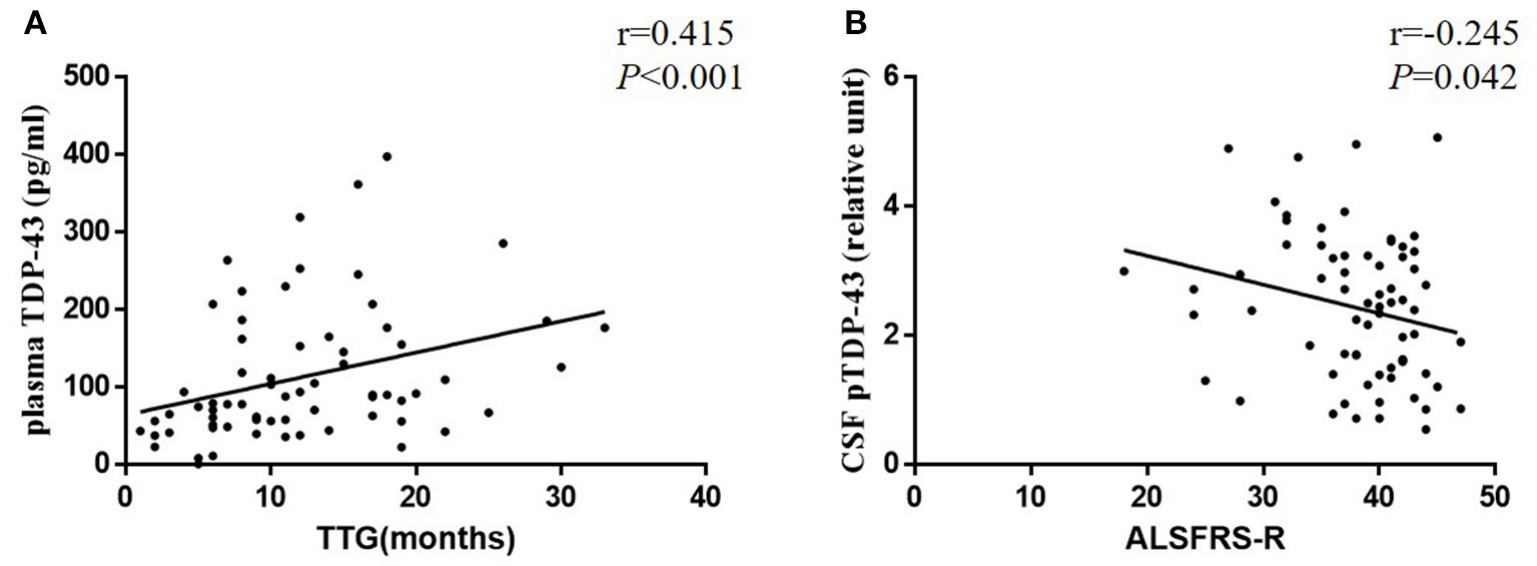
Scatter plots comparing plasma TDP-43 levels with TTG **(A)** and CSF pTDP-43 levels with age of onset **(B)** in each ALS patient.

In ALS patients, no significant difference was observed between males and females in TDP-43 (plasma: *P* = 0.410, CSF: *P* = 0.462) and pTDP-43 (plasma: *P* = 0.223, CSF: *P* = 0.800) levels. Moreover, no significant difference was noted between bulbar onset and spinal onset in ALS patients in TDP-43 (plasma: *P* = 0.179, CSF: *P* = 0.357) and pTDP-43 (plasma: *P* = 0.373, CSF: *P* = 0.159) levels (Table 2). Moreover, a correlation was observed between CSF pTDP-43 levels and the ALSFRS-R (*r* = −0.245, *P* = 0.042) (Figure 3B); Moreover, the correlation between plasma and CSF TDP-43 was observed in each ALS patient (*r* = 0.195, *P* = 0.027), however, no correlation was found between plasma or CSF pTDP-43 levels and other clinical indicators (Supplementary Material 2).

**Table 2:** Plasma and CSF TDP-43/pTDP-43 concentrations in different groups.

**Subgroup items**	**TDP-43 levels (pg/ml)**		**pTDP-43 levels (relative unit)**		***P*-value**
	**Plasma**	**CSF**	**Plasma (%)**	**CSF (%)**	***P1*/*P2*/*P*3/*P*4**
**Group**					
ALS (*n* = 69)	82.98 (102.65)	29.67 (18.05)	1.19 (0.65)	2.45 (1.77)	<0.001/0.086/ <0.001/0.065
HC (*n* = 59)	23.09 (15.23)	25.75 (24.25)	0.58 (0.55)	1.91 (1.97)	
**Gender in ALS Group**					
Male (*n* = 46)	83.82 (102.04)	29.80 (19.56)	1.16 (0.66)	2.46 (1.19)	0.410/0.462/0.223/0.800
Female (*n* = 23)	82.98 (121.09)	26.98 (17.49)	1.32 (0.74)	2.39 (1.01)	
**Site of onset in ALS group**					
Bulbar (*n* = 10)	77.02 (68.32)	32.36 (22.21)	1.16 (0.42)	1.97 (0.85)^*^	0.179/0.357/0.373/0.159
Spinal (*n* = 59)	88.41 (108.86)	29.67 (17.90)	1.19 (0.71)	2.52 (1.16)^*^	

*Data are expressed as median (IQR)*.

* *Data are expressed as mean (SD)*.

*CSF, cerebrospinal fluid; ALS, amyotrophic lateral sclerosis; HC, healthy control. P1 and P2 represent the statistically significant of TDP-43 in plasma and CSF between subgroups which expressed as P-value, respectively. P3 and P4 represent the statistically significant of pTDP-43 in plasma and CSF between subgroups which expressed as P-value, respectively*.

All ALS patients, who participated in our study, were followed up for the outcome of the disease. The mean TTG was 12.03 (range 1–33) months. No significant difference was observed between the bulbar onset (mean, 8.60 months; range 1–22 months) and spinal onset (mean, 12.61 months; range, 2–33 months) in TTG (*P* = 0.100). Moreover, a correlation was established between plasma TDP-43 levels and TTG (*r* = 0.415, *P* < 0.001), which indicated that high levels of plasma TDP-43 correlated with a long TTG (Figure 3A).

## Discussion

The presence of cytoplasmic inclusions positive for ubiquitin is a characteristic feature of degenerating motor neurons in ALS. Neumann et al. reported that TAR DNA-binding protein 43 (TDP-43), the RNA processing protein, was the major component of cytoplasmic inclusions (22). However, only little is known about the mechanism that leads to the aggregation of TDP-43 and neurodegeneration. Mutations in *TARDBP*, the TDP-43 gene, have been identified in a small number of ALS patients and account for ~7% of familial and ~2% of sporadic ALS cases (23, 24). Approximately, 15% of ALS cases develop FTD, that is, pure motor deficits may coexist with cognitive deficits compatible with FTD, which is well-recognized as the frontotemporal spectrum disorder of ALS (ALS-FTSD) (25–27). TDP-43-positive protein inclusions have been detected in majority cases of tau-negative FTD, providing the pathological basis for the clinical overlap between ALS and FTD (28).

In this study, we observed a significant increase in the levels of TDP-43 and pTDP-43 in plasma of the ALS group. Moreover, the pTDP-43/TDP-43 ratios in plasma were significantly higher in HCs than ALS, which revealed that plasma TDP-43/pTDP-43 levels and the pTDP-43/TDP-43 ratios in plasma appear to distinguish individuals with ALS from controls. In present study, it is of note that 10 out of 69 ALS patients with apparent decreased levels of CSF pTDP-43 had bulbar signs, and the variability may be due in part to differences between the bulbar-onset and spinal-onset clinical and pathological characteristics of ALS with neurodegeneration (29, 30), or the differences in the sequence to repair motor neuron damage. However, our results could not confirm this finding and showed no statistical significance between bulbar-onset and spinal-onset subgroups in CSF pTDP-43 in ALS (*P* = 0.159). Given the limited number of cases, further studies on a larger scale are required to better understand the pathology of ALS subtypes.

Previous studies stated that TDP-43 lesions in subpial/subependymal or perivascular localizations had been focused on the TDP-43-linked neurodegeneration, which might account for increased blood or CSF TDP-43 levels through mechanisms remains unclarified (31). Furthermore, the rapid neurodegeneration in ALS may release intracellular TDP-43 from inclusions, which might lead to increased TDP-43 levels in biofluids. According to the views of Feneberg et al. the quantification of TDP-43 in CSF appears limited by very low concentrations or low binding affinity of the antibodies in the presence of high abundant immunoglobulines and albumin (3). However, the question regarding the higher levels of CSF pTDP-43 in ALS patients remains unanswered. We speculate that the discrepancy could be explained by one or more of the following hypotheses: (1) higher levels of CSF pTDP-43 might be related to the more widespread pTDP-43 pathology in brain and spinal cord, which suggests that extracellular pTDP-43 could be more abundant in CSF than plasma. (2) CSF pTDP-43 levels might depend on the stage of disease or severity and release into the extracellular space by an unconventional secretory pathway which was different with TDP-43. Further longitudinal studies based on repeat sampling from the same ALS patient in the different disease stages could then be compared. (3) The permeation rate and the level of permeability of TDP-43/pTDP-43 through the damaged BBB/BSCB could potentially be different. (4) As TDP-43 is ubiquitously expressed (32) and is found in blood plasma (2), may combine with a higher level of insoluble TDP-43 in CSF, which could partly explain the relatively higher CSF pTDP-43. Previous studies showed that blood–brain barrier (BBB) and blood–spinal cord barrier (BSCB) integrity could be compromised in ALS patients. Meanwhile, evidence suggested that structural and functional damages in BBB/BSCB in the early stage of animal models (33–37). These changes might accelerate the permeation of pTDP-43 from CSF to blood, or accompanied by a extravasation of pTDP-43 due to the leaking BBB/BSCB. Further studies involving the biological or functional of pTDP-43 will address this matter.

Several cohorts investigated the TDP-43 in biofluids were rather large and included various degenerative disease groups such as ALS and FTD, and the TDP-43 levels in CSF and plasma varied among the studies, which might be attributed to the difference in experimental methods, sample collections, and storage methods. However, information about the genetic background was absent in most of the studies, since the report of pathogenic missense mutations in the *TARDBP* gene in ALS cases, which demonstrated that defects in this gene were sufficient to cause familial ALS and sporadic ALS partially (23). Hasegawa et al. developed a ALS mouse model with a mutant TDP-43 knock in (KI) that heterozygously expressed the mutant human *TARDBP* gene (A382T or G348C). They found that the TDP-43 mRNA levels in white blood cells (WBCs) of A382T mutant mice were significantly higher than that of the G348C mutant. The mRNA levels of both apoptosis-related factors (Smn1 and Naip5) correlated with the TDP-43 levels in WBCs and also differed between A382T and G348C, which suggested that each mutation in *TARDBP* induces distinct RNA metabolism and abnormal RNA metabolism is one of the causes of neuronal cell death in ALS. The study also indicate that different mutation in *TARDBP* produces varying levels of TDP-43 in WBCs (38). Therefore, the concentrations of TDP-43 in plasma and CSF could be varied in ALS patients, especially in those harboring the mutation in *TARDBP*. A previous study described the CSF TDP-43 levels in ALS patients with hexanucleotide repeat expansion (HRE) in the *C9orf72* gene, however, no difference in the CSF TDP-43 levels was observed between the *C9orf72* expansion carriers and noncarriers in the cohort (11). Given the potential consequences of gene heterogeneity, nine patients with ALS carrying pathogenic variants were excluded in our study, hitherto, there has been no relevant research concerning the impact of the *TARDBP* mutation on the levels of TDP-43 in CSF and plasma in ALS patients. Hence, large-scale and well-controlled studies, especially including subjects with gene-confirmed ALS patients are imperative.

In the current study, the AUC value was 0.924 for plasma TDP-43 levels, which generated a high sensitivity and specificity. However, a low AUC value in CSF vs. plasma in present study. We speculate three possibilities from the difference. In present study, CSF was collected into polypropylene tubes and stored in screw-cap Eppendorf tubes, and blood was collected into EDTA tubes and stored in screw caps polypropylene tubes. EDTA is a chelating agent that inhibits protease activity by binding with mental ions such as calcium ions, magnesium ions and iron ions. Recent studies showed some evidence of physiological electrolytes induced reversible aggregation of YFP-tagged full-length TDP-43 (yTDP-43) *in vitro* model system. The order of aggregation induction potency was K+ < Na+ < Mg2+ < Ca2+(39). The EDTA-plasma tubes cause less aggregation of TDP-43 than polypropylene-CSF tubes, which is consistent with the results of higher levels of TDP-43 in the plasma but little in the CSF. These differences may explain the low AUC value in CSF. A second possibility is that TDP-43 could permeate the damaged BBB/BSCB freely. However, if protease could not permeate the BBB/BSCB, or only a small minority could, then the TDP-43 levels in CSF and plasma could be different. Under the assumption of degradation and metabolism of TDP-43 in CSF is influenced by the protease, a possible hypothesis is that compared with the protease-rich CSF, TDP-43 in the plasma may be more robust and less susceptible to proteases. In addition, Feneberg et al. found that the TDP-43 CSF to blood concentration ratio is about 1:200 and TDP-43 in CSF originating mainly from blood (40), and on this basis the quantification of TDP-43 appears confined to extremely low binding affinity or concentrations in CSF (3), which presenting another possibility. In the current cohort, we used the previously published pTDP-43 ELISA method (9) to estimate its sensitivity and applied this method to evaluate the levels of pTDP-43 in CSF and plasma from ALS patients and HCs. Moreover, TTG, as an early progression parameter of ALS, was evaluated and suggested a significant correlation with plasma TDP-43 levels, which indicated that plasma TDP-43 might be optimal for the assessment of disease progression. ALSFRS-R is commonly used to reflect disease severity in ALS and a negative correlation between CSF pTDP-43 and ALSFRS-R score was observed. However, these numerical associations may simply be fortuitous, or may reflect that the data we have presented here, although intriguing, is still very much preliminary, and a large-scale prospective study would be essential to substantiate the current findings.

This is the first study comprising of an Chinese population with screened genetic background of the patient and simultaneous evaluation of the plasma and CSF TDP-43/pTDP-43 levels in ALS patients. The study showed plasma TDP-43 may play an important role in disease diagnosis and as a progression predictor of ALS. However, several caveats should be kept in mind when evaluating our findings. First, the GGGGCC copy number and expansion within the *C9orf72* gene was not evaluated in ALS patients allowing for an extremely low incidence of mutation among Chinese populations in our earlier limited understanding (41). Second, the study did not set up the disease control groups, particularly given the expected differential deposition of TDP-43/pTDP-43 in other TDP-43 proteinopathy (42), which was a disadvantage during differential diagnosis. Thirdly, the pathological assessment and confirmation of correlation between biofluid levels and protein deposition in the cerebrum or spinal cord were lacking.

## Data Availability Statement

The raw data supporting the conclusions of this article will be made available by the authors, without undue reservation.

## Ethics Statement

The studies involving human participants were reviewed and approved by the Ethics Committee of the Chinese PLA General Hospital. The patients/participants provided their written informed consent to participate in this study.

## Author Contributions

YR: concept, data analysis, experimental studies, and manuscript preparation. XH: design, manuscript editing, and manuscript review. FC: literature search and manuscript editing. ML and HW: data acquisition. FY: data analysis. XS: data analysis and statistical analysis. SC: data acquisition and data analysis. SL: experimental studies and literature search. All authors contributed to the article and approved the submitted version.

## Conflict of Interest

The authors declare that the research was conducted in the absence of any commercial or financial relationships that could be construed as a potential conflict of interest.
