# Supplementary material for: TDP-43 and Phosphorylated TDP-43 Levels in Paired Plasma and CSF Samples in Amyotrophic Lateral Sclerosis
Source: Front Neurol. 2021 Jun 14;12:663637. doi: 10.3389/fneur.2021.663637 (PMC8236522; doi:10.3389/fneur.2021.663637)
Supplement: Supplementary file 1 [file Data_Sheet_1.docx]

**Supplementary material 1. Gene information for the ALS patients**

| ALS | Gene | Amino acid changes | PolyPhen-2 | SIFT | Mutation Taster | variant frequency in ExAC |
| --- | --- | --- | --- | --- | --- | --- |
| 03 | *SETX* | SETX:NM_015046,exon5,c.431A>G,p.N144S | pathogenic | pathogenic | benign | 0.0001818 |
| 36 | *DCTN1* | DCTN1:NM_001135041,exon21,c.2783G>A,p.G928D;DCTN1,NM_0 | pathogenic | pathogenic | pathogenic | 0.000008237 |
| 37 | *DCTN1* | DCTN1:NM_001135041,exon26,c.3382G>A,p.E1128K;DCTN1,NM_ | pathogenic | benign | pathogenic | 0.0001074 |
| 40 | *SOD1* | SOD1:NM_000454,exon2,c.113G>T,p.G38V | pathogenic | pathogenic | pathogenic | - |
| 50 | *OPTN* | OPTN:NM_021980, exon13, c.1546G>C, p.E516Q | pathogenic | benign | benign | 0.00002472 |
| 55 | *FIG4* | FIG4:NM_014845,exon18,c.2063A>G,p.D688G | pathogenic | pathogenic | pathogenic | - |
| 61 | *DAO* | DAO:NM_001917,exon3,c.308C>T,p.P103L | pathogenic | benign | pathogenic | 0.0003626 |
| 66 | *DCTN1* | DCTN1:NM_001135041,exon26,c.3343G>T,p.A1115S | pathogenic | benign | pathogenic | 0.00009889 |
| 74 | *SOD1* | SOD1:NM_000454,exon5,c.420C>A,p.N140K | pathogenic | pathogenic | pathogenic | - |

ExAC= Exome Aggregation Consortium

**Supplementary material 2.**

**Correlations between plasma and CSF TDP-43/pTDP-43 levels and clinical indicators**

|  | ALSFRS-R | Disease duration | Age of onset | Progression rate | TTG | BMI | TDP43_plasma_/  pTDP-43_plasma_ |
| --- | --- | --- | --- | --- | --- | --- | --- |
|  |  |  | (*r, P)* |  |  |  |  |
| TDP-43_plasma_ | -0.040, 0.743 | 0.039, 0.750 | 0.094, 0.443 | -0.014, 0.908 | 0.415, 0.000 | 0.145, 0.234 | / |
| TDP-43_CSF_ | 0.120, 0.325 | -0.120, 0.325 | -0.135, 0.267 | 0.097, 0.428 | 0.144, 0.238 | 0.033, 0.789 | 0.195, 0.027* |
| pTDP-43_plasma_ | 0.017, 0.887 | 0.003, 0.977 | -0.025, 0.841 | 0.030, 0.804 | 0.129, 0.289 | 0.024, 0.845 | / |
| pTDP-43_CSF_ | -0.245, 0.042 | 0.010, 0.938 | 0.221, 0.068 | 0.144, 0.237 | -0.096, 0.430 | -0.012, 0.921 | 0.147, 0.097** |

ALSFRS-R=amyotrophic lateral sclerosis functional rating scale revised

CSF=cerebrospinal fluid, TTG=time to generalization

BMI=body mass index

* represents the correlation between plasma and CSF TDP-43 which expressed as r and *P* values.

** represents the correlation between plasma and CSF pTDP-43 which expressed as r and *P* values.

**Supplementary material 3. Detailed characteristics of controls**

| Case | Gender | Age | Neurological complaints | Suspected diagnosis | Final diagnosis |
| --- | --- | --- | --- | --- | --- |
| 1 | M | 40 | Limbs numbness | PN | SD |
| 2 | M | 48 | Headache | Headache | Migraine |
| 3 | M | 46 | Headache and fever | Meningitis | URI |
| 4 | F | 57 | Tinnitus and vertigo | PCI | Vertigo |
| 5 | M | 54 | Limbs numbness | PN | CS |
| 6 | M | 23 | Headache | Headache | Migraine |
| 7 | M | 76 | Vertigo and tinnitus | PCI | Vertigo |
| 8 | M | 57 | Headache | Headache | Cluster headache |
| 9 | M | 47 | Limbs numbness | PN | SD |
| 10 | M | 64 | Limbs numbness | PN | CS |
| 11 | M | 33 | Headache and vomiting | Meningitis | Venous sinus stenosis |
| 12 | M | 55 | Limbs twitches | PNHS | Muscle cramp |
| 13 | M | 48 | Headache | Headache | Migraine |
| 14 | F | 63 | Limbs twitches | PNHS | Muscle cramp |
| 15 | F | 39 | Vertigo and tinnitus | Vertigo | BPPV |
| 16 | M | 43 | Headache and vomiting | Headache | Migraine |
| 17 | F | 65 | Visual changes | ON | Maculopathy |
| 18 | F | 53 | Headache and dizziness | Headache | Hypertention |
| 19 | F | 53 | Headache | Headache | Migraine |
| 20 | M | 53 | Tinnitus and headache | Headache | TTH |
| 21 | F | 48 | Headache | Headache | Migraine |
| 22 | F | 53 | Anxiety and insomnia | Anxiety state | Anxiety state |
| 23 | F | 31 | Visual changes and headache | Headache | Migraine |
| 24 | M | 55 | Limbs numbness | PN | CS |
| 25 | M | 48 | Headache and dizziness | Headache | TTH |
| 26 | M | 61 | Dizziness and weakness | CVD | Hypertension |
| 27 | F | 65 | Agitation and insomnia | AE | Anxiety state |
| 28 | M | 64 | Limbs numbness | PN | LS |
| 29 | M | 43 | Paresthesia | PN | SD |
| 30 | F | 62 | Personality change | AE | Anxiety and depression |
| 31 | F | 58 | Headache | Headache | TTH |
| 32 | F | 38 | Headache | Headache | Migraine |
| 33 | M | 43 | Transient loss of consciousness | Syncope | Syncope |
| 34 | M | 54 | Headache and blurred vision | Headache | Glaucoma |
| 35 | F | 62 | Limbs numbness | PN | CS |
| 36 | M | 41 | Headache | Headache | Migraine |
| 37 | M | 55 | Headache | Headache | TTH |
| 38 | M | 50 | Headache | Headache | TTH |
| 39 | M | 66 | Limbs weakness and pain | PN | LS |
| 40 | F | 48 | Limbs numbness | PN | CS |
| 41 | F | 53 | Limbs numbness | PN | SD |
| 42 | F | 31 | Headache | Headache | Migraine |
| 43 | M | 57 | Limbs numbness | PN | CS |
| 44 | M | 48 | Transient loss of consciousness | Epilepsy | Syncope |
| 45 | M | 61 | Transient loss of consciousness | TIA | Syncope |
| 46 | M | 65 | Limbs weakness | MND | LS |
| 47 | M | 63 | Limbs numbness | PN | LS |
| 48 | M | 43 | Paresthesia | PN | SD |
| 49 | F | 62 | Limbs numbness | PN | CS |
| 50 | M | 58 | blurred vision | ON | glaucoma |
| 51 | F | 38 | Limbs numbness | PN | SD |
| 52 | M | 53 | Paresthesia | PN | SD |
| 53 | F | 45 | Paresthesia and anxiety | PN | SD |
| 54 | F | 53 | Limbs numbness and pain | PN | CS |
| 55 | M | 31 | Headache | Headache | Migraine |
| 56 | M | 57 | Headache | Headache | TTH |
| 57 | M | 48 | Transient loss of consciousness | Syncope | Syncope |
| 58 | M | 61 | Limbs weakness and numbness | PN | CS |
| 59 | F | 65 | Limbs weakness | MND | CS |

PN=peripheral neuropathy, SD=somatoform disorder, ON=optic neuropathy

TTH=tension-type headache, MND=motor neuron disease, CS=cervical spondylosis

BPPV=Benign paroxysmal positional vertigo, PNHS=peripheral nerve hyperexcitability syndromes

#### URI=upper respiratory infection, PCI=posterior circulation ischemia, CVD=cerebrovascular disease, LS=lumbar spondylosis, TIA=t[ransient ischemic attacks](javascript:;), AE=autoimmune encephalitis
